# Validation of a UHPLC-MS/MS Method to Quantify Twelve Antiretroviral Drugs within Peripheral Blood Mononuclear Cells from People Living with HIV

**DOI:** 10.3390/ph14010012

**Published:** 2020-12-25

**Authors:** Amedeo De Nicolò, Alice Ianniello, Micol Ferrara, Valeria Avataneo, Jessica Cusato, Miriam Antonucci, Elisa De Vivo, Catriona Waitt, Andrea Calcagno, Alice Trentalange, Giampiero Muccioli, Stefano Bonora, Giovanni Di Perri, Antonio D'Avolio

**Affiliations:** 1 Department of Medical Sciences, University of Turin, Laboratory of Clinical Pharmacology and Pharmacogenetics, Amedeo di Savoia Hospital, 10149 Turin, Italy; alice.ianniello89@gmail.com (A.I.); micol.ferrara29@gmail.com (M.F.); valeria.avataneo@unito.it (V.A.); jessica.cusato@unito.it (J.C.); miriam.antonucci20@gmail.com (M.A.); elisa.devivo59@gmail.com (E.D.V.); andrea.calcagno@unito.it (A.C.); alicetrenta@gmail.com (A.T.); stefano.bonora@unito.it (S.B.); giovanni.diperri@unito.it (G.D.P.); antonio.davolio@unito.it (A.D.); 2 Department of Molecular and Clinical Pharmacology, University of Liverpool, Liverpool L69 7BE, UK; C.Waitt@liverpool.ac.uk; 3 Department of Drug Science and Technology, University of Turin, 10149 Turin, Italy; giampiero.muccioli@unito.it

**Keywords:** liquid chromatography, tandem mass spectrometry, peripheral blood mononuclear cells, dolutegravir, intracellular

## Abstract

Recently, anti-HIV treatment has achieved high efficacy and tolerability. Nevertheless, few data are available about the intracellular penetration of antiretrovirals, partly due to the technical challenges related to intracellular quantification. This work aimed to validate an ultra-high performance liquid chromatography (UHPLC) tandem mass spectrometry (MS/MS) method for the simultaneous quantification of maraviroc, nevirapine, rilpivirine, dolutegravir, raltegravir, cobicistat, darunavir, ritonavir, atazanavir, efavirenz, elvitegravir, and etravirine within peripheral blood mononuclear cells (PBMCs) and apply it to samples from patients. PBMCs were isolated by density gradient on cell preparation tubes (CPT). Samples were prepared by addition of internal standards (IS), sonication, centrifugation, and drying. Reconstituted extracts underwent chromatographic separation by reversed phase UHPLC and detection was performed by electrospray ionization and multiple reaction monitoring. Method validation followed FDA and EMA guidelines, showing acceptable accuracy, precision, recovery and IS-normalized matrix effect. The application to 56 samples from patients undergoing antiretroviral treatment provided description of intracellular penetration, showing method eligibility for future studies.

## 1. Introduction

Nowadays, combination antiretroviral therapy (cART), consisting of the combination treatment with several antiretroviral drugs (ARVs) from at least two different ARV classes, has considerably improved tolerability, efficacy, and genetic barrier to resistance, greatly increasing life expectancy in people living with HIV (PLWH) [1]. The major classes of currently available ARVs include nucleoside reverse transcriptase inhibitors (NRTIs), non-nucleoside reverse transcriptase inhibitors (NNRTIs), protease inhibitors (PIs), entry inhibitors (EI), fusion inhibitors (FI), and integrase strand-transfer inhibitors (INSTI) [2,3,4]. Currently, the standard cART entails the administration of two NRTIs as a “backbone” and at least one drug from another class, while in recent years some “nucleoside-sparing” regimens are also being investigated and adopted, combining at least two highly potent drugs from two different classes [5,6]. In particular, dolutegravir (DTG) has recently been identified as a first-line treatment for HIV infection in adults and adolescents [7]. Despite the current effectiveness of cART in suppressing HIV viral load, HIV infection remains a chronic condition, due to latency and residual replication of the provirus in viral reservoirs [8,9,10,11,12,13,14]. These include lymphoid tissues, lymph nodes, the central nervous system and latent infected lymphocytes and monocytes [9,10,14,15].

It is important to note that current monitoring of plasma concentrations of ARVs does not predict their penetration into the target cells and, consequently, into the reservoirs [16]. In recent years, several studies have been conducted to determine the penetration of ARVs into peripheral blood mononuclear cells (PBMCs), used as a surrogate for the viral reservoirs [9,17,18,19]. It has been demonstrated that ARV concentrations in mononuclear cells extracted from different compartments (e.g., peripheral blood, lymph node, and rectal lymphoid tissue) can differ according to the molecule, but these are usually considerably lower than in plasma, leading to tissue-specific underdosing [9,20]. These findings greatly increased the interest in studying intracellular pharmacokinetics (PK). Moreover, the recent introduction and development of cell-targeted prodrugs (e.g., tenofovir-alafenamide) [21] or nanoformulations [22,23] further support the importance of studying intracellular ARV PK. Nevertheless, to date, the study of intracellular concentrations has been limited to a small number of studies, mainly due to technical challenges and invasiveness in isolating PBMCs or obtaining tissue biopsies, respectively, as well as the need for very high analytical sensitivity. Therefore, the intracellular quantification of ARVs is not widely applied in research, while application in clinical practice, such as for therapeutic drug monitoring (TDM), remains a remote prospect.

The current technical challenges in this field include PBMC isolation, cell counting, preparation and analysis by liquid chromatography and tandem mass spectrometry [9,24,25,26,27]. In fact, PBMC isolates have widely variable cell numbers, purity, and activation state [28], affecting both data normalization [29] and chromatographic signal [30].

Therefore, thoroughly validated methods for the quantification of ARVs in PBMCs are needed in order to estimate their penetration in viral reservoirs. Several methods have been reported for the quantification of ARVs in PBMCs [25,27,31,32] and in other cell types [33], but these included only a few currently used ARVs; moreover, these methods mainly focused on NRTIs [21]. Furthermore, there are no reported methods for the simultaneous intracellular quantification of dolutegravir (DTG), elvitegravir (ELV), and rilpivirine (RPV), together with other ARVs of non-nucleos(t)ide nature.

In this work, a fast and reliable ultra-high performance liquid chromatography (UHPLC) tandem mass spectrometry (MS/MS) method has been developed, validated following FDA and EMA guidelines [34,35], and applied to a small cohort of samples from PLWH under treatment with several different ARV regimens. The compounds investigated in this work included NNRTIs (nevirapine, NVP, etravirine, ETV, efavirenz, EFV, and RPV), PIs (darunavir, DRV, and atazanavir, ATV), PK boosters (cobiscistat, COBI, and ritonavir, RTV), INSTI (raltegravir, RAL, ELV, and DTG), and an EI (maraviroc, MVC).

## 2. Results and Discussion

### 2.1. Calibration Curve and Dilution Integrity

Mean determination coefficients (*R*^2^) of all calibration curves ranged from 0.996 to 0.999 (Table 1), with a linear regression model forced through zero. The low concentrations and the presence of SIL-ISs limited the saturation phenomenon. Dilution of samples over the Upper Limit Of Quantification ULOQ led to inaccuracy and imprecision lower than 15%.

### 2.2. Specificity and Selectivity

**Figure 1:**
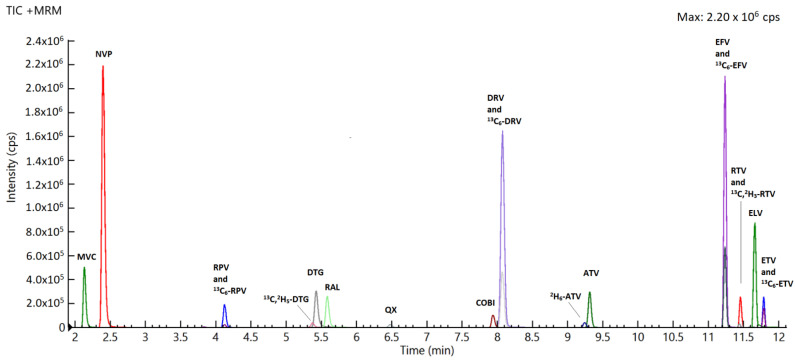
Representative chromatogram of a chemical mix at concentration of 2 μg/mL for each drug.

The assay did not show any significant interference with tested drugs. Analytes’ retention times are summarized in Table 1 and an overlaid chromatogram is reported in Figure 1.

The blank PBMC samples did not show any endogenous interference causing a significant “noise”, taking into account the retention times of the analytes.

### 2.3. Accuracy and Precision

The validation results in terms of accuracy and precision are listed in Table 2. Both imprecision and inaccuracy were below 15%, in keeping with FDA and EMA guidelines [34,35].

### 2.4. Lowest Limit of Quantification (LLOQ) and Limit of Detection (LOD)

The determination of the LOD was carried out as reported in Section 2.8, executing serial dilutions of the lowest calibrator, standard 1 (STD1) for each drug. LLOQ and LOD are shown in Table 1.

**Figure 2:**
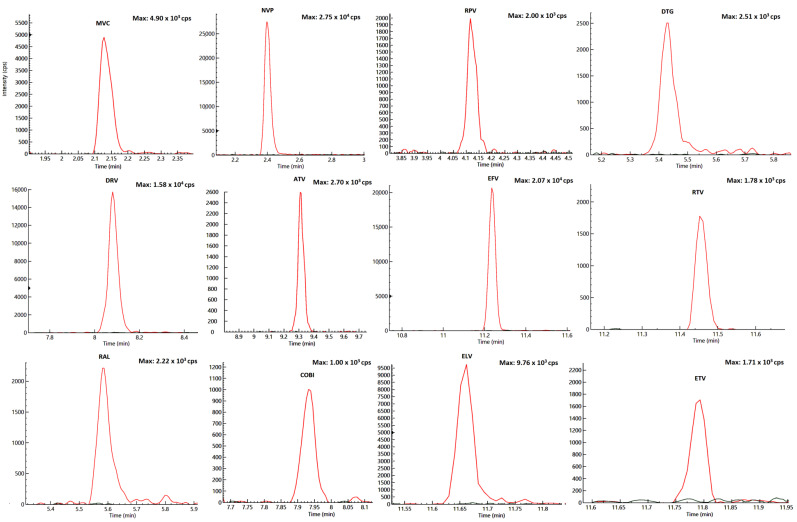
Overlaid chromatograms of blank peripheral blood mononuclear cells (PBMC) (STD 0) and LOD (limit of detection) for each drug.

The LLOQ, investigated in five replicates (as other quality control (QC) samples), was at least equal to the lowest point of the curve (STD1) as required by the FDA guidelines, without significant interfering peaks by sample matrix (Figure 2).

### 2.5. Recovery

Recovery data are summarized in Table 3, demonstrating consistency and reproducibility for each compound, as well as concordance between target analytes and their ISs. The lower recovery values for COBI, ATV, and ETV were supposed to be due to a possible adsorption to the cellular matrix but considering that their relative standard deviation (RSD)% was contained, these were considered acceptable.

### 2.6. Matrix Effect

Matrix effect (ME) data are summarized in Table 3. Despite the fact that the mean values of ME were high in some cases, particularly for COBI and ELV, their RSD% was within 15%, thus being acceptable according to EMA guidelines and previous reports [30,34]. The mean values of IS-nME were generally lower than the ones of ME, indicating that the ISs were able to reduce the mean ME. Nevertheless, their corrective impact on the RSD% values was not always appreciable (e.g., NVP, DRV, and ELV), still remaining within the 15% limit.

### 2.7. Carry-Over

Carry-over experiments led to signals which were lower than at the LOD. The mean IS area obtained from the analysis of blank PBMC samples was lower than 1% of the one observed in PBMC samples containing IS, confirming the absence of significant carry-over.

### 2.8. Stability

All drugs were stable bench-top for 24 h (25 °C with artificial light), showing a degradation below 7% at each concentration. All the analytes and IS, kept for 24 h in the autosampler at 10 °C, showed a variation lower than 6% at each concentration.

Taking into account the analytical variability, the processed samples were stable throughout the UHPLC/MS-MS analysis, always completed within 24 h.

### 2.9. Testing of Patients’ Samples

The new method was tested on 56 PBMC samples from 30 HIV positive patients undergoing cART mainly based on boosted PIs. Concentrations were evaluated in samples taken at the pharmacological steady state, at the end of the inter-dose interval (trough concentrations) and with detectable drug concentrations in plasma. These samples corresponded to 19 different ARV combinations, including all the drugs considered in this method, except for EFV and NVP. Among these, 34 contained DTG, 2 RAL, 1 ELV, 1 RPV, 5 ETV, 31 DRV, 21 ATV, 29 RTV, and 17 COBI. The quantification of each drug was successful in all samples, showing concentrations within the calibration ranges (Table 4). Concentrations for all drugs were comparable to previously published data [36]. Concerning intra-PBMC/plasma ratios, DTG exhibited the lowest intra-PBMC penetration, followed by RAL, DRV, ATV, MVC, COBI, RTV, RPV, ETV, and ELV (Table 4). The IntraPBMC/plasma ratios were significantly and moderately correlated (*R*^2^ = 0.391; *p*-value = 0.025) to the LogP values of each drug.

## 3. Materials and Methods

### 3.1. Chemicals

Pure reference standard powders of NVP, EFV, RTV, COBI, MVC, RPV, DTG, RAL, ATV, DRV, ELV, and ETV (chemical structures are depicted Figure S1) were purchased from Clinisciences (Milan, Italy), with a minimum purity of 98%; 6,7-Dimethyl- 2,3-di(2-pyridyl) quinoxaline (QX, purity 99.9%), used as an internal control of instrument performance, was purchased from Sigma-Aldrich (Milan, Italy); Stable-Isotope-Labeled ARVs used as Internal Standards (SIL-IS), including ^2^H_6_-ATV (purity 98%, isotopic enrichment 98%), ^13^C_6_-RPV (purity 96.4%, isotopic enrichment 99%, used as IS for MVC, NVP and RPV), ^13^C_6_-DRV (purity 100%, isotopic enrichment 99.4%, used as IS for DRV and COBI), ^13^C,^2^H_5_-DTG (purity 95.5%, isotopic enrichment 99.3% and 99.7%, for ^13^C and ^2^H, used as IS for DTG and RAL), ^13^C_6_-EFV (purity 98.2%, isotopic enrichment 99%), ^13^C_6_-ETV (purity 99.3%, isotopic enrichment 99%, used as IS for ETV and ELV), and ^13^C,^2^H_3_-RTV (purity 100%, isotopic enrichment 99%), were purchased from Alsachim (Illkirch Graffenstaden, France). The positions of the labels are shown in Figure S2. HPLC grade acetonitrile and methanol were purchased from VWR International (Radnor, PA, USA). HPLC grade water was produced with Milli-DI system coupled with a Synergy 185 system by Millipore (Milan, Italy). Formic acid (FA) was purchased from Sigma-Aldrich (Milan, Italy).

Buffy coats for blank PBMC isolation and plasma from healthy donors were kindly supplied by the Blood Bank of the “Città della Salute e della Scienza” of Turin.

### 3.2. Stock Solutions, Standards, and Quality Controls

DRV, NVP, MVC, ATV, and RAL stock solutions were prepared in a solution of methanol and HPLC grade water (90:10 *v*:*v*), while EFV, ETV, RTV, COBI, RPV, ELV, and DTG were dissolved in a solution of methanol and HPLC grade water (95:5 *v*:*v*) at 1 mg/mL; all stock solutions were then refrigerated at −20 °C until use, within 1 month.

A working solution of IS was made by diluting QX (20 ng/mL), ^2^H_6_-ATV, ^13^C_6_-RPV, ^13^C,^2^H_5_-DTG and ^13^C_6_-ETV (at 25 ng/mL), ^13^C_6_-DRV and ^13^C_6_-EFV (at 250 ng/mL) and ^13^C,^2^H_3_-RTV (10 ng/mL), in methanol and HPLC grade water (70:30 *v*:*v*), and orthophosphoric acid 0.005% at each analytical session.

Calibration standards (STDs) and quality controls (QCs) were prepared by spiking “blank” PBMC aliquots with 100 µL of previously prepared “spiking solutions”. The highest spiking solution (to prepare STD 9) and the ones for the preparation of three QCs (High, Medium, and Low) were prepared at 100 ng/mL, 80 ng/mL, 10 ng/mL, and 1 ng/mL, except for NVP, DRV, and EFV, which were prepared at a 10-fold higher concentration, by directly diluting stock solutions with water:acetonitrile 70:30 (*v*:*v*); the other spiking solutions were prepared by serial (1:1) dilution of the highest one, to obtain nine different concentrations. Drug concentrations in the spiking solutions and the final absolute amounts in the STD samples, respectively, are listed in Table 1.

The absolute amounts of drugs in the calibration standards were 0.039 ng, 0.078 ng, 0.156 ng, 0.313 ng, 0.625 ng, 1.250 ng, 2.500 ng, 5.000 ng, and 10.000 ng for STDs 1, 2, 3, 4, 5, 6, 7, 8, and 9, respectively, except for NVP, DRV, and EFV, which had 10-fold higher amounts. All the solutions and samples were stored at −80 °C, for no more than six months until analysis, avoiding more than three freeze–thaw cycles.

### 3.3. Chromatographic Conditions

The chromatographic system consisted of a LX-50 UHPLC (Perkin Elmer). The chromatographic separation was performed using an Acquity^®^ UPLC HSS T3 column, 2.1 × 150 mm, 1.8 μm (Waters, Milan), maintained at 50 °C. Autosampler temperature was set at 10 °C.

Flow rate was 0.4 mL/min, with a gradient of two mobile phases (MP): MP-A (0.05% *v*:*v* FA in water) and MP-B (0.05% *v*:*v* FA in acetonitrile). Briefly, the chromatographic gradient started with 70:30 MP-A:MP-B for 0.3 min, then the percentage of MP-B linearly increased to 47%, 55%, 60%, 75%, and 95% at 8, 9, 10, 11, and 11.7 min, respectively, achieving the separation and elution of all the analytes. The column was washed with 95% MP-B for 1.5 min and reconditioned for 1.8 min, for a total runtime of 15 min.

Two different autosampler washing solutions were used: a “weak-washing solution” (water:acetonitrile 70:30 *v*:*v*) and a “strong-washing solution” (water:acetonitrile 30:70 *v*:*v*). Two “strong” washing steps and 3 “weak” washing steps (250 µL each) were applied after each injection. Plasma concentrations were evaluated by the same analytical method, following a sample preparation protocol described by Simiele et al. [37]. Plasma concentration data underwent internal and external quality assessment (INSTAND, EQA, Dusseldorf, Germany), with conforming results.

### 3.4. Mass Spectrometry Conditions

Tandem mass spectrometric detection was carried out with a QSight^®^ 220 (Perkin Elmer, Milan, Italy) tandem mass spectrometer, with electrospray ionization (ESI) interface. The ESI source was set in positive ionization mode (ESI+) for all drugs except for EFV, which was detected by negative electrospray ionization (ESI−).

“Zero-Air” (dry air) was used as a nebulizing and heating gas, while nitrogen was used as “drying and collision” gas: both these gases were produced at high purity (>99.9%) with a Zefiro ^®^ HQ (Cinel, Vigonza, Italy) gas generator.

MS conditions were optimized by direct infusion of reference standards for each drug (100 ng/mL in MP-A:MP-B 50:50 *v*:*v*) at 10 μL/min, combined with a mobile phases MP-A:MP-B (50:50 *v*:*v*) flow at 0.4 mL/min from the analytical column.

General mass parameters for positive ionization were: electrospray voltage 5.5 kV; source temperature 350 °C; nebulizing gas flow 350 L/h; heating gas flow 350 L/h; drying gas flow 130 L/h; heated surface induced desolvation (HSID) temperature 270 °C. Two mass transitions yielding the highest sensitivity were selected for each drug and quantification was performed using multiple reaction monitoring (MRM), summarized in Table 1. One transition was used as a quantification trace, while the other one was used as confirmation (secondary ion trace). Mass data were acquired and processed through Simplicity^®^ software ver. 4.6.3 (Perkin Elmer, Milan, Italy).

### 3.5. PBMC Isolation

PLWH receiving several cART regimens containing the target drugs at standard doses and who gave informed consent, as indicated by the local Ethical Committee approvals, were enrolled in the context of the approved clinical study ENHANCERS (Prot. N° 1479/007A/2017, 24/11/2017); a protocol aiming to evaluate the effect of pharmacokinetic boosters of plasma and intracellular ARV concentrations (COBI, RTV, or no booster). These patients underwent blood sampling at the end of the dosing interval (C_trough_), with cell preparation tubes (CPT^®^, Becton, Dickinson and Co., Franklin Lakes, NJ, USA, for PBMC isolation) and with lithium heparin tubes (for plasma). Plasma was obtained after centrifugation at 1400× *g* (3000 rpm) for 10 min at 4 °C (Jouan Centrifuge, Model BR4i, Saint-Herblain, France). It then underwent heat inactivation, drug extraction, and analysis as previously described [37]. Blood samples (2 CPT of 8 mL each, total volume 16 mL) from patients were processed following a PBMC isolation protocol previously described in several studies [38,39,40,41].

Briefly, CPTs were centrifuged at 1700× *g* for 15 min at 20 °C, then the PBMC layer was transferred to Falcon tubes and adjusted at the volume of 40 mL with NaCl 0.9% (isotonic) solution. After a centrifugation at 700× *g* for 6 min at 4 °C, the supernatant was discarded and the resulting pellet was washed with 2 mL of an ammonium salts solution (3.5 g ammonium chloride + 0.036 g ammonium carbonate in 500 mL of water) for 1 min, in order to achieve the lysis of residual erythrocytes. Then the solution was diluted to a final volume of 40 mL with NaCl 0.9%: two aliquots of 500 μL were further diluted 1:40 with NaCl 0.9% (final volume 20 mL) and used to perform cell counts and mean cell volume (MCV) measurement with an automated Z2 Beckman Coulter (Instrumentation Laboratory, Milan, Italy). The remaining 39 mL of solution were immediately centrifuged at 700*× g* for 6 min at 4 °C and the supernatant was discarded. All the washing solutions were kept at 4 °C, in order to reduce any drug efflux during cell isolation.

Cell pellets from patients were resuspended with 1 mL of water:methanol 30:70 (*v*:*v*), divided in two aliquots (500 μL) and stored at −80 °C until analysis. The number of cells and MCV data for each sample from patients were used to determine the total volume of cells in each aliquot, allowing the conversion of results from “ng/aliquot” to “ng/mL”, as previously described [41].

Blank PBMCs used for the preparation of standards and quality controls were extracted from buffy coats, following the same protocol used for isolation from blood.

The “blank” PBMC isolates were resuspended in water:methanol 30:70 (*v*:*v*) and stored at a concentration of 16 × 10^6^ cells/aliquots.

### 3.6. STDs, QCs, and Patient Samples Extraction

Blank PBMC samples (500 µL each) were spiked with 100 µL of the corresponding spiking solutions, in order to obtain the STD and QC samples; similarly, STD 0 and patient samples were spiked with 100 µL of water:acetonitrile 70:30 (*v*:*v*), in order to mimic the STDs volume. Each sample was then spiked with 40 µL of IS working solution, and then, after vortex-mixing for 10 s, it underwent sonication for 10 min at room temperature. Subsequently, samples were centrifuged at 21,000× *g* for 10 min, without brake, at 4 °C.

Supernatants were then transferred to glass tubes and dried in a vacuum centrifuge at 50 °C (nearly 1.5 h). Dry extracts were resuspended with 100 µL of water:acetonitrile 70:30 (*v*:*v*) and 10 µL were injected in the chromatographic system. Statistical treatment of analytical data was performed through Microsoft Office Excel (ver. 2007) and SPSS 26.0 (IBM, Armonk, NY, USA).

### 3.7. Specificity and Selectivity

Interference from endogenous compounds was investigated by analysis of six different blank PBMC samples, at medium and high cell numbers (16 × 10^6^ and 8 × 10^6^ cells/aliquot).

Potential interference by co-medications administered to the patients was evaluated by spiking blank PBMC samples with them. These included NRTIs (zidovudine, lamivudine, abacavir, tenofovir, emtricitabine, and tenofovir alafenamide), anti-tubercular drugs (ethambutol, isoniazid, pyrazinamide, rifampicin, and rifabutin), antibiotics and antimycotics (caspofungin, ceftazidime, ciprofloxacin, moxifloxacin, levofloxacin, linezolid, piperacillin, tazobactam, ceftriaxone, daptomycin, isavuconazole, fluconazole, posaconazole, voriconazole, and itraconazole).

“Interference” was defined as observable ion suppression/enhancement or cross-talk with any of the target analytes.

### 3.8. Accuracy, Precision, Calibration, and Limit of Quantification

Intra-day and inter-day precision and accuracy were determined by analyzing three different QC concentrations (plus the lowest limit of quantification, LLOQ) in six validation sessions.

Accuracy was calculated as the ratio between the mean analytical result and the nominal concentration. Inter-day and intra-day imprecision were expressed as the relative standard deviation (RSD%) at each QC concentration, at the three different QC samples (and at the LLOQ) between sessions and in 5 replicates in the same analytical session, respectively. A maximum RSD% value of 15% and a minimum mean accuracy value of 85% was considered as acceptable.

Each calibration curve was obtained by processing the nine STD points in double replicate.

Calibration curves were processed by analytes’ peak area normalized by areas of IS compounds.

A “linear through zero” with 1/x weighting regression model was used for all curves, and model fitting was evaluated up to a concentration twice as high as STD 9. The forced through 0 model was chosen in order to highlight eventual lack of linearity at low levels and to avoid an “overweighting” of the low standard points for the definition of calibration curves (calibration parameters are shown in Table S1). The back-calculated concentrations of at least 7 out of 9 standard samples had to be within a 15% of bias, as suggested by EMA and FDA guidelines, in order to accept the calibration curve (mean accuracy of STD in Table S2).

Dilution integrity was tested by analyzing five replicates of a sample spiked at a concentration twice as high as STD 9 (which was considered as the ULOQ), after a 3-fold dilution with blank PBMC lysates from healthy donors.

Limit of detection (LOD) was defined as the concentration that yielded a signal-to-noise ratio of at least 3:1. “Noise” was evaluated as the mean MS signal at the analytes’ retention times corresponding to the injection of a “blank” PBMC sample. Percentage of deviation from the nominal concentration (measure of accuracy) and relative standard deviation (measure of precision) of the concentration considered as the lower limit of quantification (LLOQ) had to be < 20%, and it was considered as the lowest calibration standard, as suggested by FDA and EMA guidelines [34,35].

### 3.9. Recovery

Recovery from PBMC samples was assessed by comparing the peak areas obtained from multiple analyses of QC samples (High, Medium, and Low) with peak areas obtained from the injection of blank PBMC extracts spiked after the extraction (post-extraction addition method) at the same concentrations.

### 3.10. Stability

Stability data for the considered drugs were already reported in previously published studies [42,43,44,45,46,47], so no further extensive long-term stability evaluation was performed in this study. On the other hand, confirmative reanalysis of 11 patient samples containing RPV, DTG, ATV, RTV, DRV, and COBI over a period of three months indicated a maximum deviation of 8% (for RTV), which was within method variability [24,25,27,33,48,49]. Furthermore, a short-term evaluation of stability in the working conditions (in the autosampler and “bench-top”) was performed up to 24 h.

### 3.11. Matrix Effect

The “matrix effect” (ME) was investigated in six lots of blank PBMC at two different cell numbers (16 × 10^6^ and 8 × 10^6^ cells/aliquot) from different donors, as suggested by the guidelines [34,35]. Peak areas from blank extracts spiked with all analytes at three QC concentrations were compared with peak areas from standard solutions (prepared in water:acetonitrile 70:30 *v*:*v*) spiked at the same concentrations. The ME was calculated as the percent deviation between the peak areas obtained from the PBMC extracts and those obtained from the standard solutions. Moreover, in order to estimate the capability of IS compounds for correcting ME variability, the “IS-normalized ME” was also calculated, as previously reported [30] and suggested in EMA guidelines [34].

### 3.12. Carry-Over

Carry-over was investigated in triple replicates by injecting extracted blank PBMC extracts immediately after samples containing target analytes at concentrations twice as high as STD 9. A value ≤ 20% of the lower limit of quantification (LLOQ) and a value ≤ 5% for IS were considered as absence of significant carry-over.

## 4. Conclusions

In this work, a robust, practical, and relatively economical approach has been described for the quantification of all the current and frequently used ARVs within PBMC within a single assay. This method is relatively cheap since it does not need solid phase extraction, the SIL-ISs are now widely available on the market more cheaply than previously, and the amount of IS needed for each extraction is quite low (1 mg of SIL-IS covers theoretically more than 3 × 10^5^ samples). This approach, from sampling, isolation, counting, and final analysis, has the great advantage of obtaining results expressed as concentration, being comparable with those from plasma.

By the application of this method to a small cohort of PLWH treated with a wide range of different combination treatments, several interesting data were observed, which confirmed the marked differences between plasma and intra-PBMC concentration of ARVs.

Interestingly, different classes showed opposite behaviors in terms of intracellular penetration: with the exception of DRV, PIs as well as pharmacokinetic boosters which share similar chemical properties, showed higher intracellular penetration (in accordance with previous data [18,19]), while intra-PBMC concentrations of INSTIs were found to be nearly one third of those in plasma, with the exception of ELV. These results are in accordance with the recent work from Dyavar et al. [50], evidencing a rather poor penetration of INSTIs within lymphoid cells, with the only exception being ELV, which exhibited a very high penetration, probably due to its high LogP value.

It is worth mentioning that the correlation between LogP and intracellular penetration was moderate (*R*^2^ = 0.391), highlighting that it is not the only leading force at the basis of intracellular disposition of ARVs: other important factors, such as drug transporters (e.g., P-gP), are already known to have an impact on intracellular penetration of ARVs, as well as underlying some of its inter-individual variability [51,52,53].

Nevertheless, despite the interesting information about ARV penetration within PBMCs, this study presents some limitations, which should be taken into account. Firstly, the heterogeneous range of regimens enabled simply a description of intracellular penetration of these drugs in a “real life” setting, whereas comparison between regimens was impossible due to the low numbers of samples. Nevertheless, this type of analysis will surely be the object of future studies.

Secondly, although this method allows a reliable quantification of ARVs within the cells, it cannot verify the real disposition of drugs within the cell: in fact, a portion of these drugs, which are quite lipophilic, could be bound to the cell membranes or organelles [17]. For this reason, the direct use of intracellular concentrations of ARVs for pharmacodynamic evaluations might potentially result in an overestimation of their antiviral effect. From a practical point of view, the use of 2 CPT tubes (16 mL) which was chosen in order to ensure a rugged analysis even in potentially lymphocytopenic patients, could be problematic for a large scale application or for particularly critical patients. Nonetheless, the observed concentrations in patients’ samples were always abundantly within the calibration ranges, indicating that, probably, the analysis could be successfully conducted starting from a lower blood volume (1 single CPT, 8 mL).

Notwithstanding these limitations, the intracellular quantification of ARVs in PBMC provides an important marker of drug penetration into lymphocytes and monocytes, as well as a better surrogate of the quantification in lymphoid tissues than plasma. The method presented in this work, validated to FDA and EMA standards, will enable such analysis in a greater number of future clinical studies.

## Figures and Tables

**Table 1:** For each compound are reported, in order: mean retention time (RT), highest spiking solution concentration, final drug amounts in the standard (STD) 9 to STD1/LLOQ, LOD, dwell times and mass transitions (parent ions, first and second daughter ions), with the corresponding cone voltages and collision energies. Unit “ng/sample” refers to the absolute amount of drug added to a blank PBMC aliquot of 16 × 10^6^ cells, to obtain the calibration STDs. LLOQ: lowest limit of quantification.

Drugs	RT (min)	Highest Spiking Solution (ng/mL)	STD9/ULOQ (ng/Sample)	STD1/LLOQ (ng/Sample)	LOD (ng/Sample)	*R* ^2^	[M-H] + (*m*/*z*)	Dwell (ms)	Entrance Voltage (V)	FIRST Trace (*m*/*z*)	Collision Energy First Ion Trace (eV)	Second Trace (*m*/*z*)	Collision Energy Second Ion Trace (eV)
MVC	2.15	100	10	0.039	0.019	0.996	514.4	30	33	117.2	−69	389.3	−26
NVP	2.35	1000	100	0.390	0.195	0.997	267.1	30	25	80.2	−56	226.1	−32
RPV	4.08	100	10	0.039	0.019	0.998	367.3	30	45	128.2	−83	195.2	−48
DTG	5.32	100	10	0.039	0.019	0.998	420.3	25	30	127.1	−46	277.1	−34
RAL	5.60	100	10	0.039	0.019	0.996	445.2	25	26	109.1	−41	361.1	−25
COBI	7.90	100	10	0.039	0.019	0.996	776.4	25	15	98.1	−82	606.2	−34
DRV	8.15	1000	100	0.390	0.195	0.998	548.3	25	15	69.1	−66	392.2	−18
ATV	9.30	100	10	0.039	0.019	0.999	705.4	30	10	144.2	−59	168.15	−58
EFV *	11.21	1000	100	0.390	0.195	0.997	314.1	25	−29	68.9	48	244.1	22
RTV	11.44	100	10	0.039	0.019	0.996	721.4	25	27	140.1	−95	296.15	−29
ELV	11.64	100	10	0.039	0.019	0.996	448.2	25	30	143.0	−61	344.0	−42
ETV	11.76	100	10	0.039	0.019	0.997	435.1	25	51	77.1	−110	144.1	−55
^13^C_6_-RPV	3.67	-	1	1	1	-	373.3	30	39	182.2	−50	195.2	−50
^13^C_,_^2^H_5-_DTG	5.32	-	1	1	1	-	426.3	25	40	133.2	−50	277.1	−35
QX	6.48	-	0.8	0.8	0.8	-	313.1	25	44	78.1	−72	246.2	−43
^13^C_6_-DRV	8.15	-	10	10	10	-	554.3	25	16	113.1	−31	398.2	−20
^2^H_6_-ATV	9.21	-	1	1	1	-	711.5	30	39	147.2	−61	338.2	−41
^13^C_6_-EFV *	11.17	-	10	10	10	-	320.1	25	−24	68.9	61	250.1	23
^13^C_,_^2^H_3_-RTV	11.35	-	0.4	0.4	0.4	-	725.3	25	8	201.1	−57	272.2	−41
^13^C_6_-ETV	11.72	-	1	1	1	-	441.2	25	45	77.1	−110	150.2	−55

* Negative ionization.

**Table 2:** Summary of quality controls (QCs) final amount, accuracy, intra-day, and inter-day precision (relative standard deviation, RSD) for all drugs.

Drugs	QC High					QC Medium					QC Low				
	Spiking Sol. Conc. (ng/mL)	Final Amount per Sample (ng)	Accuracy %	Imprecision RSD%		Spiking Sol. Conc. (ng/mL)	Final Amount per Sample (ng)	Accuracy %	Imprecision RSD%		Spiking Sol. Conc. (ng/mL)	Final Amount per Sample (ng)	Accuracy %	Imprecision RSD%	
				Intra-Day %	Inter-Day %				Intra-Day %	Inter-Day %				Intra-Day %	Inter-Day %
MVC	80	8	95.6	5.3	11.5	10	1	95.5	3.4	14.7	1	0.1	92.5	6.0	7.6
NVP	800	80	96.1	4.0	10.0	100	10	104.8	1.9	12.0	10	1	87.7	5.8	9.2
RPV	80	8	103.7	2.8	5.2	10	1	87.4	4.4	3.2	1	0.1	89.7	4.5	10.6
DTG	80	8	98.6	2.9	7.6	10	1	85.1	3.4	13.8	1	0.1	93.4	7.2	6.8
RAL	80	8	106.3	4.1	6.0	10	1	90.8	4.3	9.9	1	0.1	92.8	5.9	9.5
COBI	80	8	94.7	7.8	8.5	10	1	86.9	7.7	12.0	1	0.1	95.2	1.5	9.3
DRV	800	80	101.4	1.8	3.7	100	10	87.4	3.0	9.5	10	1	88.1	3.3	12.0
ATV	80	8	103.7	2.7	5.2	10	1	92.7	3.4	12.9	1	0.1	94.4	6.1	9.3
EFV	800	80	103.1	1.1	3.7	100	10	90.8	3.1	5.7	10	1	88.1	4.4	4.0
RTV	80	8	111.5	12.6	9.5	10	1	93.5	12.1	9.7	1	0.1	94.4	9.9	8.2
ELV	80	8	100.4	4.7	8.4	10	1	95.5	3.0	5.3	1	0.1	98.9	8.7	7.9
ETV	80	8	104.1	1.8	4.0	10	1	90.1	4.5	9.5	1	0.1	94.3	5.5	5.9

**Table 3:** Mean recovery and mean matrix effect for all drugs.

Drugs	Recovery (RSD)	Matrix Effect (RSD)	IS-Normalized Matrix Effect (RSD)
MVC	89.2% (6.1%)	4.6% (6.7%)	13.9% (9.1%)
NVP	89.5% (2.6%)	−10.1% (5.9%)	−2.4% (10.5%)
RPV	83.2% (3.0%)	−5.0% (11.6%)	2.8% (4.7%)
DTG	92.8% (3.8%)	−11.1% (7.2%)	0.1% (4.7%)
RAL	91.2% (2.2%)	−15.7% (5.4%)	−5.3% (4.9%)
COBI	77.6% (3.0%)	31.2% (9.1%)	19.8% (7.5%)
DRV	84.6% (2.9%)	−15.0% (4.3%)	−1.0% (12.9%)
ATV	80.3% (2.9%)	9.3% (6.0%)	−0.1% (4.2%)
EFV	86.6% (2.4%)	−4.0% (4.8%)	−0.6% (1.7%)
RTV	85.3% (2.7%)	−8.1% (7.7%)	−7.2% (8.0%)
ELV	87.3% (4.3%)	−27.4% (10.3%)	−10.1% (10.5)
ETV	79.7% (3.1%)	−17.6% (6.0%)	4.5% (6.6%)

**Table 4:** Summary of the median observed concentrations in patients’ plasma and PBMC and their relative penetration in PBMC.

	Median Trough Concentrations (IQR)			
Drugs	*n*	Plasma (ng/mL)	PBMC (ng/mL)	PBMC/Plasma Ratio
MVC	1	68 (na *)	163 (na)	2.40 (na)
RPV	1	80 (na)	399 (na)	4.98 (na)
DTG	34	913 (616–1392)	270 (156–450)	0.26 (0.14–0.53)
RAL	2	138 (136–na)	43 (40–na)	0.31 (0.29–na)
COBI	17	49 (6–142)	204 (63–686)	3.52 (3.15–4.48)
DRV	31	2389 (1523–3963)	935 (581–1642)	0.34 (0.23–0.57)
ATV	21	778 (424–1547)	1429 (552–3255)	1.53 (1.01–2.72)
RTV	29	82 (47–389)	497 (278–845)	3.81 (2.07–6.70)
ELV	1	228 (na)	1641 (na)	7.20 (na)
ETV	5	514 (78–763)	2137 (500–3126)	5.51 (3.01–6.03)

* na = not applicable.

## Data Availability

Validation data are available in supplementary materials and within the text. Application data are available on request due to privacy and ethical restrictions.

## Supplementary Materials

The following are available online at https://www.mdpi.com/1424-8247/14/1/12/s1, Figure S1: Chemical Structures of the target analytes, Figure S2: Chemical structures and labeling of the stable-isotope linked internal standards. Table S1: calibration curves parameters for each analyte. Actual intercepts were set to 0 due to the adoption of a “linear through zero” with 1/x weighing calibration model. Each final model has to be considered as: “Response = Slope * concentration + 0”. The mean observed intercept removing “through zero forcing” is also reported, for comparison, Table S2: Mean accuracy percentages of the back-calculated concentrations of calibration standards during the validation sessions.

## References

[1] Younai F.S. (2013). Thirty years of the human immunodeficiency virus epidemic and beyond. Int. J. Oral Sci..

[2] Rathbun R.C., Lockhart S.M., Miller M.M., Liedtke M.D. (2014). Dolutegravir, a second-generation integrase inhibitor for the treatment of HIV-1 infection. Ann. Pharmacother..

[3] Shah B.M., Schafer J.J., Desimone J.A. (2014). Dolutegravir: A new integrase strand transfer inhibitor for the treatment of HIV. Pharmacotherapy.

[4] WHO (2008). Updated Recommendations on First-Line and Second-Line Antiretroviral Regimens and Post-Exposure Prophylaxis and Recommendations on Early Infant Diagnosis of HIV: Interim Guidelines.

[5] Orkin C., Llibre J.M., Gallien S., Antinori A., Behrens G., Carr A. (2017). Nucleoside reverse transcriptase inhibitor-reducing strategies in HIV treatment: Assessing the evidence. HIV Med..

[6] Murray J.M., Emery S., Kelleher A.D., Law M., Chen J., Hazuda D.J., Nguyen B.Y., Teppler H., Cooper D.A. (2007). Antiretroviral therapy with the integrase inhibitor raltegravir alters decay kinetics of HIV, significantly reducing the second phase. AIDS.

[7] Panel on Antiretroviral Guidelines for Adults and Adolescents Guidelines for the Use of Antiretroviral Agents in Adults and Adolescents with HIV. Department of Health and Human Services. http://www.aidsinfo.nih.gov/ContentFiles/AdultandAdolescentGL.pdf.

[8] Rochat M.A., Schlaepfer E., Kuster S.P., Li D., Audige A., Ivic S., Fahrny A., Speck R.F. (2018). Monitoring HIV DNA and cellular activation markers in HIV-infected humanized mice under cART. Virol. J..

[9] Fletcher C.V., Staskus K., Wietgrefe S.W., Rothenberger M., Reilly C., Chipman J.G., Beilman G.J., Khoruts A., Thorkelson A., Schmidt T.E. (2014). Persistent HIV-1 replication is associated with lower antiretroviral drug concentrations in lymphatic tissues. Proc. Natl. Acad. Sci. USA.

[10] Lorenzo-Redondo R., Fryer H.R., Bedford T., Kim E.Y., Archer J., Pond S.L.K., Chung Y.S., Penugonda S., Chipman J., Fletcher C.V. (2016). Persistent HIV-1 replication maintains the tissue reservoir during therapy. Nature.

[11] Rothenberger M.K., Keele B.F., Wietgrefe S.W., Fletcher C.V., Beilman G.J., Chipman J.G., Khoruts A., Estes J.D., Anderson J., Callisto S.P. (2015). Large number of rebounding/founder HIV variants emerge from multifocal infection in lymphatic tissues after treatment interruption. Proc. Natl. Acad. Sci. USA.

[12] Svicher V., Ceccherini-Silberstein F., Antinori A., Aquaro S., Perno C.F. (2014). Understanding HIV compartments and reservoirs. Curr. HIV/AIDS Rep..

[13] Bon I., Calza L., Musumeci G., Longo S., Bertoldi A., D’Urbano V., Gibellini D., Magistrelli E., Viale P.L., Re M.C. (2017). Impact of Different Antiretroviral Strategies on Total HIV-DNA Level in Virologically Suppressed HIV-1 Infected Patients. Curr. HIV Res..

[14] Chargin A., Yin F., Song M., Subramaniam S., Knutson G., Patterson B.K. (2015). Identification and characterization of HIV-1 latent viral reservoirs in peripheral blood. J. Clin. Microbiol..

[15] Moron-Lopez S., Puertas M.C., Galvez C., Navarro J., Carrasco A., Esteve M., Manye J., Crespo M., Salgado M., Martinez-Picado J. (2017). Sensitive quantification of the HIV-1 reservoir in gut-associated lymphoid tissue. PLoS ONE.

[16] Nagano D., Araki T., Yanagisawa K., Ogawa Y., Gohda F., Uchiumi H., Handa H., Nakamura T., Yamamoto K. (2018). Darunavir concentration in PBMCs may be a better indicator of drug exposure in HIV patients. Eur. J. Clin. Pharmacol..

[17] Dyavar S.R., Ye Z., Byrareddy S.N., Scarsi K.K., Winchester L.C., Weinhold J.A., Fletcher C.V., Podany A.T. (2018). Normalization of cell associated antiretroviral drug concentrations with a novel RPP30 droplet digital PCR assay. Sci. Rep..

[18] De Nicolò A., Simiele M., Calcagno A., Abdi A.M., Bonora S., Di Perri G., D’Avolio A. (2014). Intracellular antiviral activity of low-dose ritonavir in boosted protease inhibitor regimens. Antimicrob. Agents Chemother..

[19] D’Avolio A., Simiele M., Calcagno A., Siccardi M., Larovere G., Agati S., Baietto L., Cusato J., Tettoni M., Sciandra M. (2013). Intracellular accumulation of ritonavir combined with different protease inhibitors and correlations between concentrations in plasma and peripheral blood mononuclear cells. J. Antimicrob. Chemother..

[20] Mitchell C., Roemer E., Nkwopara E., Robbins B., Cory T., Rue T., Fletcher C.V., Frenkel L. (2014). Correlation between plasma, intracellular, and cervical tissue levels of raltegravir at steady-state dosing in healthy women. Antimicrob. Agents Chemother..

[21] Podany A.T., Bares S.H., Havens J., Dyavar S.R., O’Neill J., Lee S., Fletcher C.V., Swindells S., Scarsi K.K. (2018). Plasma and intracellular pharmacokinetics of tenofovir in patients switched from tenofovir disoproxil fumarate to tenofovir alafenamide. AIDS.

[22] Perazzolo S., Shireman L.M., Koehn J., McConnachie L.A., Kraft J.C., Shen D.D., Ho R.J.Y. (2018). Three HIV Drugs, Atazanavir, Ritonavir, and Tenofovir, Coformulated in Drug-Combination Nanoparticles Exhibit Long-Acting and Lymphocyte-Targeting Properties in Nonhuman Primates. J. Pharm. Sci..

[23] Kraft J.C., McConnachie L.A., Koehn J., Kinman L., Collins C., Shen D.D., Collier A.C., Ho R.J. (2017). Long-acting combination anti-HIV drug suspension enhances and sustains higher drug levels in lymph node cells than in blood cells and plasma. AIDS.

[24] Ter Heine R., Hillebrand M.J., Rosing H., van Gorp E.C., Mulder J.W., Beijnen J.H., Huitema A.D. (2009). Quantification of the HIV-integrase inhibitor raltegravir and detection of its main metabolite in human plasma, dried blood spots and peripheral blood mononuclear cell lysate by means of high-performance liquid chromatography tandem mass spectrometry. J. Pharm. Biomed. Anal..

[25] ter Heine R., Davids M., Rosing H., van Gorp E.C., Mulder J.W., van der Heide Y.T., Beijnen J.H., Huitema A.D. (2009). Quantification of HIV protease inhibitors and non-nucleoside reverse transcriptase inhibitors in peripheral blood mononuclear cell lysate using liquid chromatography coupled with tandem mass spectrometry. J. Chromatogr. B Analyt. Technol. Biomed. Life Sci..

[26] Pelerin H., Compain S., Duval X., Gimenez F., Benech H., Mabondzo A. (2005). Development of an assay method for the detection and quantification of protease and non-nucleoside reverse transcriptase inhibitors in plasma and in peripherical blood mononuclear cells by liquid chromatography coupled with ultraviolet or tandem mass spectrometry detection. J. Chromatogr. B Analyt. Technol. Biomed. Life Sci..

[27] Nagano D., Araki T., Nakamura T., Yamamoto K. (2013). Determination of intracellular darunavir by liquid chromatography coupled with fluorescence detection. J. Chromatogr. Sci..

[28] Corkum C.P., Ings D.P., Burgess C., Karwowska S., Kroll W., Michalak T.I. (2015). Immune cell subsets and their gene expression profiles from human PBMC isolated by Vacutainer Cell Preparation Tube (CPT) and standard density gradient. BMC Immunol..

[29] Simiele M., D’Avolio A., Baietto L., Siccardi M., Sciandra M., Agati S., Cusato J., Bonora S., Di Perri G. (2011). Evaluation of the mean corpuscular volume of peripheral blood mononuclear cells of HIV patients by a coulter counter to determine intracellular drug concentrations. Antimicrob. Agents Chemother..

[30] De Nicolò A., Cantu M., D’Avolio A. (2017). Matrix effect management in liquid chromatography mass spectrometry: The internal standard normalized matrix effect. Bioanalysis.

[31] Becher F., Pruvost A., Gale J., Couerbe P., Goujard C., Boutet V., Ezan E., Grassi J., Benech H. (2003). A strategy for liquid chromatography/tandem mass spectrometric assays of intracellular drugs: Application to the validation of the triphosphorylated anabolite of antiretrovirals in peripheral blood mononuclear cells. J. Mass Spectrom..

[32] Belkhir L., De Laveleye M., Vandercam B., Zech F., Delongie K.A., Capron A., Yombi J., Vincent A., Elens L., Haufroid V. (2015). Quantification of darunavir and etravirine in human peripheral blood mononuclear cells using high performance liquid chromatography tandem mass spectrometry (LC-MS/MS), clinical application in a cohort of 110 HIV-1 infected patients and evidence of a potential drug-drug interaction. Clin. Biochem..

[33] Patel S.H., Ismaiel O.A., Mylott W.R., Yuan M., Hauser K.F., McRae M. (2019). Simultaneous determination of intracellular concentrations of tenofovir, emtricitabine, and dolutegravir in human brain microvascular endothelial cells using liquid chromatography-tandem mass spectrometry (LC-MS/MS). Anal. Chim. Acta.

[34] EMA Guideline on Bioanalytical Method Validation. http://www.ema.europa.eu/docs/en_GB/document_library/Scientific_guideline/2011/08/WC500109686.pdf.

[35] FDA Guidance for Industry: Bioanalytical Method Validation. http://www.fda.gov/downloads/drugs/guidancecomplianceregulatoryinformation/guidances/ucm368107.pdf.

[36] Ford N., Lee J., Andrieux-Meyer I., Calmy A. (2011). Safety, efficacy, and pharmacokinetics of rilpivirine: Systematic review with an emphasis on resource-limited settings. HIV AIDS (Auckl.).

[37] Simiele M., Ariaudo A., De Nicolo A., Favata F., Ferrante M., Carcieri C., Bonora S., Di Perri G., De Avolio A. (2017). UPLC-MS/MS method for the simultaneous quantification of three new antiretroviral drugs, dolutegravir, elvitegravir and rilpivirine, and other thirteen antiretroviral agents plus cobicistat and ritonavir boosters in human plasma. J. Pharm. Biomed. Anal..

[38] Agnesod D., De Nicolò A., Simiele M., Mohamed Abdi A., Boglione L., Di Perri G., D’Avolio A. (2013). Development and validation of a useful UPLC-MS/MS method for quantification of total and phosphorylated-ribavirin in peripheral blood mononuclear cells of HCV+ patients. J. Pharm. Biomed. Anal..

[39] De Nicolò A., Abdi A.M., Boglione L., Baiett L., Allegra S., Di Perri G., D’Avolio A. (2015). UPLC-MS/MS method with automated on-line SPE for the isomer-specific quantification of the first-generation anti-HCV protease inhibitors in peripheral blood mononuclear cells. J. Pharm. Biomed. Anal..

[40] De Nicolò A., Agnesod D., Simiele M., Rigano D., Adriani A., Canaparo R., Astegiano M., Rizzetto M., Di Perri G., D’Avolio A. (2014). UPLC-MS/MS method for quantification of the azathioprine metabolites 6-mercaptoguanosine and 6-methylmercaptopurine riboside in peripheral blood mononuclear cells. J. Pharm. Biomed. Anal..

[41] De Nicolò A., Bonifacio G., Boglione L., Cusato J., Pensi D., Tomasello C., Di Perri G., D’Avolio A. (2015). UHPLC-MS/MS method with automated on-line solid phase extraction for the quantification of entecavir in peripheral blood mononuclear cells of HBV+ patients. J. Pharm. Biomed. Anal..

[42] Aouri M., Calmy A., Hirschel B., Telenti A., Buclin T., Cavassini M., Rauch A., Decosterd L.A. (2013). A validated assay by liquid chromatography-tandem mass spectrometry for the simultaneous quantification of elvitegravir and rilpivirine in HIV positive patients. J. Mass Spectrom..

[43] Bennetto-Hood C., Tabolt G., Savina P., Acosta E.P. (2013). A sensitive HPLC-MS/MS method for the determination of dolutegravir in human plasma. J. Chromatogr. B Analyt. Technol. Biomed. Life Sci..

[44] D’Avolio A., Baietto L., Siccardi M., Sciandra M., Simiele M., Oddone V., Bonora S., Di Perri G. (2008). An HPLC-PDA method for the simultaneous quantification of the HIV integrase inhibitor raltegravir, the new nonnucleoside reverse transcriptase inhibitor etravirine, and 11 other antiretroviral agents in the plasma of HIV-infected patients. Ther. Drug Monit..

[45] D’Avolio A., Sciandra M., Siccardi M., Baietto L., de Requena D.G., Bonora S., Di Perri G. (2007). A simple and sensitive assay for determining plasma tipranavir concentration in the clinical setting by new HPLC method. J. Chromatogr. B Analyt. Technol. Biomed. Life Sci..

[46] DeJesus E., Rockstroh J.K., Henry K., Molina J.M., Gathe J., Ramanathan S., Wei X., Yale K., Szwarcberg J., White K. (2012). Co-formulated elvitegravir, cobicistat, emtricitabine, and tenofovir disoproxil fumarate versus ritonavir-boosted atazanavir plus co-formulated emtricitabine and tenofovir disoproxil fumarate for initial treatment of HIV-1 infection: A randomised, double-blind, phase 3, non-inferiority trial. Lancet.

[47] Djerada Z., Feliu C., Tournois C., Vautier D., Binet L., Robinet A., Marty H., Gozalo C., Lamiable D., Millart H. (2013). Validation of a fast method for quantitative analysis of elvitegravir, raltegravir, maraviroc, etravirine, tenofovir, boceprevir and 10 other antiretroviral agents in human plasma samples with a new UPLC-MS/MS technology. J. Pharm. Biomed. Anal..

[48] Colombo S., Beguin A., Telenti A., Biollaz J., Buclin T., Rochat B., Decosterd L.A. (2005). Intracellular measurements of anti-HIV drugs indinavir, amprenavir, saquinavir, ritonavir, nelfinavir, lopinavir, atazanavir, efavirenz and nevirapine in peripheral blood mononuclear cells by liquid chromatography coupled to tandem mass spectrometry. J. Chromatogr. B Analyt. Technol. Biomed. Life Sci..

[49] Prathipati P.K., Mandal S., Pon G., Vivekanandan R., Destache C.J. (2017). Pharmacokinetic and Tissue Distribution Profile of Long Acting Tenofovir Alafenamide and Elvitegravir Loaded Nanoparticles in Humanized Mice Model. Pharm. Res..

[50] Dyavar S.R., Gautam N., Podany A.T., Winchester L.C., Weinhold J.A., Mykris T.M., Campbell K.M., Alnouti Y., Fletcher C.V. (2019). Assessing the lymphoid tissue bioavailability of antiretrovirals in human primary lymphoid endothelial cells and in mice. J. Antimicrob. Chemother..

[51] Jones K., Bray P.G., Khoo S.H., Davey R.A., Meaden E.R., Ward S.A., Back D.J. (2001). P-Glycoprotein and transporter MRP1 reduce HIV protease inhibitor uptake in CD4 cells: Potential for accelerated viral drug resistance?. AIDS.

[52] Khoo S.H., Hoggard P.G., Williams I., Meaden E.R., Newton P., Wilkins E.G., Smith A., Tjia J.F., Lloyd J., Jones K. (2002). Intracellular accumulation of human immunodeficiency virus protease inhibitors. Antimicrob. Agents Chemother..

[53] Lee C.G., Gottesman M.M., Cardarelli C.O., Ramachandra M., Jeang K.T., Ambudkar S.V., Pastan I., Dey S. (1998). HIV-1 protease inhibitors are substrates for the MDR1 multidrug transporter. Biochemistry.

