# Supplementary material for: Validation of a UHPLC-MS/MS Method to Quantify Twelve Antiretroviral Drugs within Peripheral Blood Mononuclear Cells from People Living with HIV
Source: Pharmaceuticals (Basel). 2020 Dec 25;14(1):12. doi: 10.3390/ph14010012 (PMC7824452; doi:10.3390/ph14010012)
Supplement: Supplementary file 1 [file pharmaceuticals-14-00012-s001.pdf]

Supplementary table 1: calibration curves parameters for each analyte. Actual intercepts were set to 0 due to the adoption of a "linear through zero" with 1/x weighing calibration model. Each final model has to be considered as: "Response = Slope * concentration + 0". The mean observed intercept removing "through zero forcing" is also reported, for comparison.

DRUGs	R^2	Slope	Slope RSD%	Intercept - without zero forcing
**MVC**	0.996	3.20	5.8	-0.014
**NVP**	0.997	6.12	6.9	0.113
**RPV**	0.998	3.76	8.2	0.025
**DTG**	0.998	1.05	5.3	0.011
**RAL**	0.996	1.33	6.4	0.013
**COBI**	0.996	0.41	7.1	0.007
**DRV**	0.998	0.34	9.2	0.031
**ATV**	0.999	0.85	8.7	0.008
**EFV**	0.997	0.11	3.6	0.022
**RTV**	0.996	5.71	4.7	0.060
**ELV**	0.996	4.95	8.3	0.010
**ETV**	0.997	1.21	4.6	0.011

Supplementary table 2: Mean accuracy percentages of the back-calculated concentrations of calibration standards during the validation sessions.

DRUGs	STD1 - LLOQ	STD2	STD3	STD4	STD5	STD6	STD7	STD8	STD9 - ULOQ
**MVC**	83.2	91.3	96.2	98.2	97.6	96.7	97.1	98.2	101.2
**NVP**	116.3	105.6	104.3	101.3	98.9	99.1	97.2	96.1	95.3
**RPV**	111.3	106.7	103.5	99.8	98.1	97.6	96.7	97.2	96.3
**DTG**	114.6	105.2	103.2	101.2	100.7	99.6	100.1	98.2	96.7
**RAL**	116.2	104.3	106.2	102.3	101.2	101.3	96.7	95.3	95.8
**COBI**	118.2	109.6	106.8	103.1	102.3	100.5	98.5	94.6	96.2
**DRV**	116.1	106.1	106.3	102.7	99.6	97.3	95.4	95.6	94.7
**ATV**	115.8	103.7	98.3	99.2	100.1	99.3	98.6	96.2	97.3
**EFV**	114.3	104.9	95.6	96.7	97.1	96.3	96.0	95.8	95.1
**RTV**	119.1	111.2	105.8	103.2	102.1	98.3	95.6	96.2	95.8
**ELV**	118.1	109.9	101.3	98.7	96.5	96.8	95.6	94.8	95.3
**ETV**	114.3	108.2	106.2	101.1	100.1	98.6	96.7	96.9	96.7

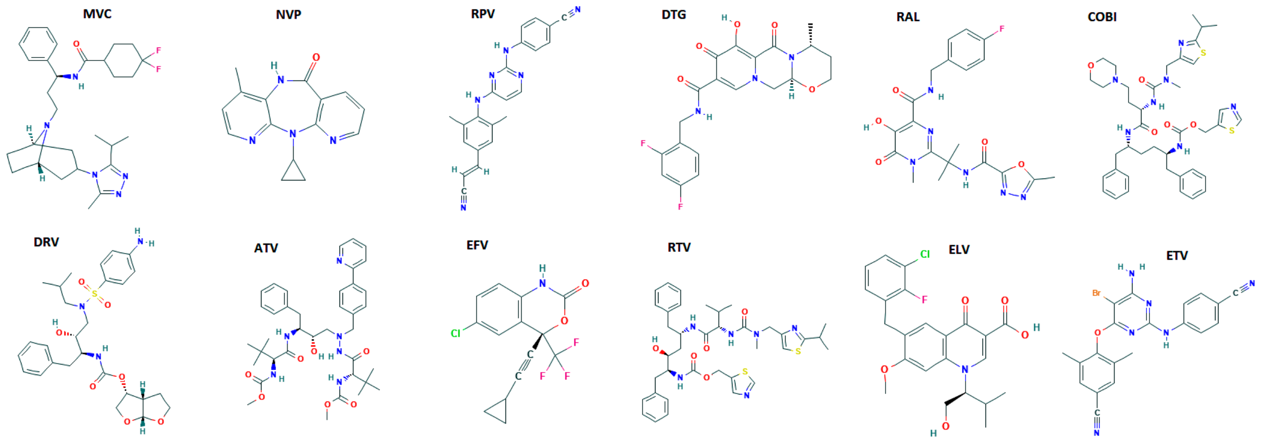

Supplementary - ARV chemical structures.

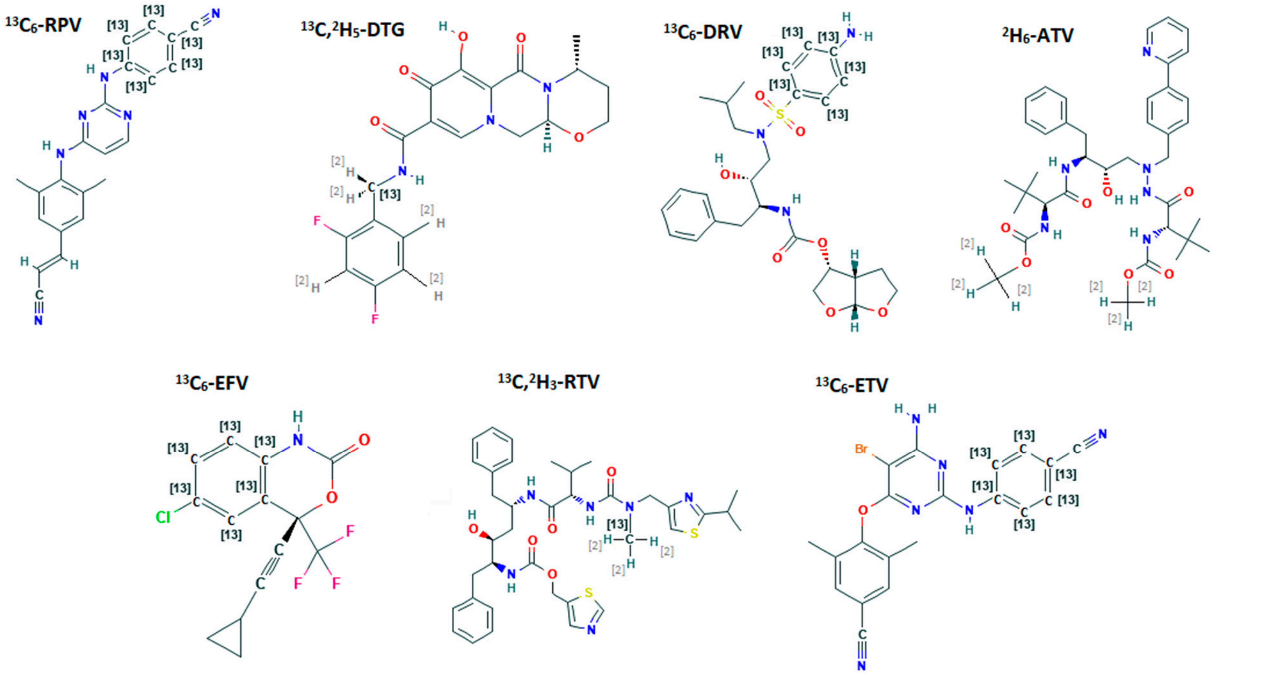

Supplementary Figure S1- SIL-ISs

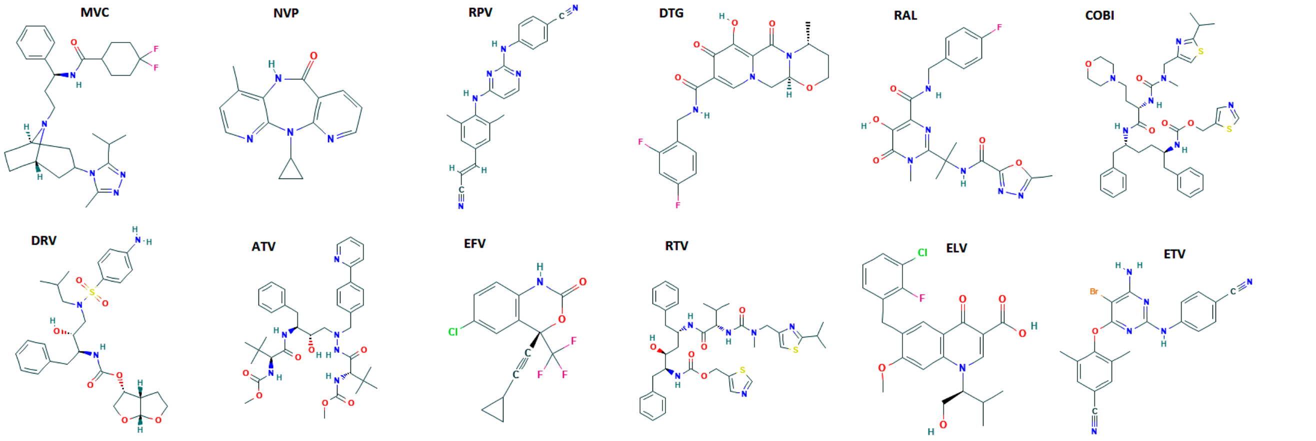

Supplementary Figure S1 - ARV chemical structures
